# Chromoanagenesis: a piece of the macroevolution scenario

**DOI:** 10.1186/s13039-020-0470-0

**Published:** 2020-01-28

**Authors:** Franck Pellestor, Vincent Gatinois

**Affiliations:** 1 Unit of Chromosomal Genetics, Department of Medical Genetics, Arnaud de Villeneuve Hospital, Montpellier CHRU, 371 avenue du Doyen Gaston Giraud, 34295 Montpellier Cedex 5, France; 2 INSERM 1183 «Genome and Stem Cell Plasticity in Development and Aging », Institute of Regenerative Medicine and Biotherapies, St Eloi Hospital, Montpellier, France

**Keywords:** Chromoanagenesis, Chromothripsis, Chromoanasynthesis, Chromoplexy, Macroevolution, Complex chromosome rearrangements, Saltational evolution, Speciation

## Abstract

Over the last decade, new types of massive and complex chromosomal rearrangements based on the chaotic shattering and restructuring of chromosomes have been identified in cancer cells as well as in patients with congenital diseases and healthy individuals. These unanticipated phenomena are named chromothripsis, chromoanasynthesis and chromoplexy, and are grouped under the term of chromoanagenesis. As mechanisms for rapid and profound genome modifications in germlines and early development, these processes can be regarded as credible pathways for genomic evolution and speciation process. Their discovery confirms the importance of genome-centric investigations to fully understand organismal evolution.

Because they oppose the model of progressive acquisition of driver mutations or rearrangements, these phenomena conceptually give support to the concept of macroevolution, known through the models of “Hopeful Monsters” and the “Punctuated Equilibrium”. In this review, we summarize mechanisms underlying chromoanagenesis processes and we show that numerous cases of chromosomal speciation and short-term adaptation could be correlated to chromoanagenesis-related mechanisms.

In the frame of a modern and integrative analysis of eukaryote evolutionary processes, it seems important to consider the unexpected chromoanagenesis phenomena.

## Background

The last decade has seen the emergence of new concepts in the field of the chromosomal mechanics and genetics, with the identification of a novel class of complex chromosome rearrangements (CCRs) arising during single cellular events and leading to massive and chaotic genomic rearrangements confined to one or a few chromosomes. The unanticipated catastrophic phenomena are named chromothripsis, chromoanasynthesis and chromoplexy, and the term “chromoanagenesis” (for chromosome rebirth) has been proposed to encompass these new types of rearrangements [1]. Although original and surprising in their formation and their complexity, these phenomena take into account previous cytogenetic data having demonstrated the respective role of recurrent and non-recurrent chromosomal abnormalities in the evolution of cancers [2]. While non-recurring abnormalities were considered as non-significant background, these studies demonstrated their link with genomic instability and karyotype evolution [3, 4].

The prevalence of theories based on gene-centric concepts has dismissed evolution scenarios based on genomic-centric concept changes [5] that the discovery of chromoanagenesis phenomena brings to light. The concept of chromoanagenesis deeply upset our designs concerning the genesis and the etiology of complex chromosomal rearrangements. It also provides new insight into the plasticity and the instability of the genome as well as on the mechanisms underlying the maintenance and the modification of chromosome structure. Accumulating data indicate that chromoanagenesis-related phenomena may occur in germlines or during early embryonic development [6] and they can lead to the formation of stable and heritable rearranged genomic constitutions [7]. Thus, as mechanism for the fast restructuring of genome, chromoanagenesis might be evolutionary relevant process. In this review, we summarize the characteristics of the 3 distinct forms of chromoanagenesis-related phenomena and we discuss their potential implications in evolutionary biology.

### All-in-one

**Fig. 1:**
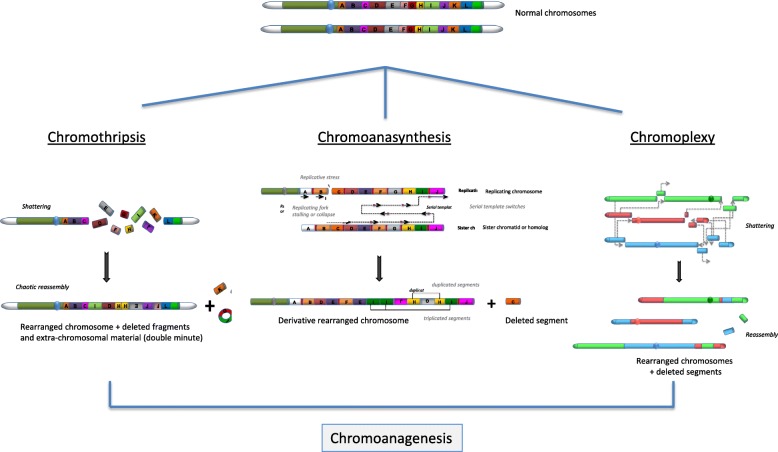
Characteristics of the chaotic mutational processes chromothripsis, chromoanasynthesis and chromoplexy, grouped under the term of chromoanagenesis. Chromothripsis refers to the localized shattering and reshuffling of one or a few chromosome segments during a one-step catastrophic event, with the incomplete repair of double-strand breaks (DSBs) through non-homologous end-joining (NHEJ). Chromoanasynthesis arise from the defective replication of a single or a few chromosomes mediated by fork-stalling and template switching (FoSTeS) or microhomology-mediated break-induced replication (MMBIR) processes. Chromoplexy involves a series of chained, complex inter- and intra-chromosome translocations including up to eight chromosomes and presumably occurring simultaneously

For each phenomenon, several specific features have been described, allowing each mechanism to be distinguished from each other (Fig. 1).

Chromothripsis (for breaking into small pieces) was the first of these chaotic processes, described in 2011 [8]. Chromothripsis results from a single cellular event in which one or several chromosomes segments are broken in pieces and reassembled in random order and orientation to form complex derivative chromosomes [9]. First observed in tumors, the phenomenon was rapidly identified in patients with congenital malformations, developmental disorders or carrying apparently balanced rearrangements [10, 11]. In the same way, chromothripsis-like events were described in phenotypically normal subjects as well as in prenatal diagnosis [12, 13].

The key-features, common to all chromothripsis events are the occurrence of numerous clustered chromosomal breakpoints, the low DNA copy number changes and the preservation of heterozygosity in the rearranged segments [14, 15]. Several mechanisms involving telomere attrition, mitotic errors, abortive apoptosis, premature chromosome condensation, p53 defect, or viral integration were identified as cellular processes driving chromothripsis [16–20]. An attractive mechanistic explanation to link all these causal processes with the confined nature of genomic alterations generated by chromothripsis, is that the implicated chromosome(s) can be sequestrated into a micronucleus in which chromothripsis-related damages will occur [21, 22]. Micronuclei formation can result from chromosome segregation failure but also can be caused by a wide variety of stresses occurring during any stages of the cell cycle [23, 24]. Micronuclei may persist in daughter cells over several cell cycles before being eliminated or reincorporated into the regular nucleus. Consequently, micronuclei must be regarded as an important source of genetic variations [25]. Experimental models have been developed making it enable to reproduce in vitro chromothripsis-like events and thus validate its existence. In particular, elegant models to specifically induce the sequestration of chromosomes Y into micronuclei and analyse their subsequent chaotic alterations have been described [19].

Chromoanasynthesis (for synthesis of new chromosomes) is a distinct form of “one-step” chaotic chromosomal rearrangement described by Liu et al. [26]. Based on DNA replication defects, chromoanasynthesis involves multiple template-switching events driven by microhomology-mediated break-induced replication (MMBIR) or fork stalling and template switching (FoSTeS) mechanisms. Disengagement of the lagging strand and annealing at a nearby replication fork site might serially occur and thus cause complex and multiple rearrangements of different scales Chromoanasynthesis can lead to the creation of complex rearranged chromosomal segments with frequent DNA copy-number changes, particularly region-focused duplications and triplications and short stretches of micro-homologies at the breakpoint junctions [27, 28].

By blocking replicative fork progression, various endogenous and exogenous factors might cause replication stress and induce genomic instability which can lead to chromoanasynthesis events [29]. Like for chromothripsis, the incorporation of lagging chromosome or chromatid fragments within micronuclei offers an appealing mechanistic explanation for chromoanasynthesis. After the cell enters S phase, DNA replication can occur in the micronuclei. However, micronuclei show significant reduction in the recruitment of components for both DNA replication and repair machinery [23]. The DNA replication in micronuclei is asynchronous and defective compared to the primary nucleus and the rupture of the micronuclear envelope alters active replication fork progression by diluting the material components of micronuclei into the cytoplasm [24].

Although the mechanism differs from that of chromothripsis, the biological consequences of chromoanasynthesis are similar, with the formation of highly rearranged chromosomes. As with chromothripsis, developmental delay, autism spectrum disorders, and dysmorphic facial features are the main disorders observed in patients with chromoanasynthesis. Chromoanasynthesis has also been observed in phenotypically unaffected individuals [30, 31].

In addition, a third type of “all-at-once” mechanism for massive chromosomal rearrangement has been evidenced in prostate cancer, and then in lung cancer and melanoma. Termed chromoplexy (for chromosome restructuring), this phenomenon is characterized by the interdependent occurrence of multiple inter- and intra-chromosomal translocations and deletions [32]. These chains of rearrangements, numbering from 3 to over 40, can involve up to 8 chromosomes in a single chain. All the translocated segments originate from DNA double-strand breaks and the derivative rearranged chromosomes present little or no copy number alterations [33]. Although chromoplexy includes multiple chromosomes, it is possible that such chain of translocations was promoted by physical clustering of the breakpoints in the nucleus, in relation with the colocalization of DNA replication or transcription factories. Since multiple chromosomes can be encapsulated in micronuclei, it is also conceivable that a micronucleus-based model could mediate the process of chromoplexy [24].

Some chromoanagenesis events arise de novo, but accumulating data on familial chromoanagenesis-mediated rearrangements have validated the notion of the heritability of chromoanagenesis-mediated genomic alterations [12, 34]. Recent reports have provided evidence that chromoanagenesis can operate in human germline cells and during early embryonic development [35–37], strongly suggesting that chromoanagenesis could be more common than anticipated in gametes and preimplantation embryos.

### Chromoanagenesis and saltational evolution

To date, numerous studies have confirmed the biological reality of chromoanagenesis events. In cancer, the discovery of these phenomena has challenged the dogma that genomic alterations in tumoral cells occur through progressive accumulation of mutational events [38]. The chromoanagenesis model offers additional routes towards tumorigenesis that the gradual route. In the field of constitutional genetics, recent reports have demonstrated that complex chromoanagenesis-related rearrangements can be viable and transmissible [39]. In the light of this data emerges a new perception of how the genome can be rapidly reworked and how chaotic rearrangements could be credible mechanisms for genome evolution while maintaining its stability. Because all chromoanagenesis-related processes are fast mechanisms occurring from a single cellular event, and they oppose the model of progressive acquisition of driver mutations, the concept of chromoanagenesis supports the evolution models that purpose long periods of relative stability punctuated by sudden and rapid periods of radical modifications.

The implication of large and rapid genetic alterations in speciation was first proposed by the geneticist R. Goldschmidt (1878–1958) who advanced a model of saltational evolution known as the “Hopeful Monster” model [40]. Goldschmidt raised no objection to the standard neo-Darwinian view of gradual accumulation of small mutations (micro-evolution) but he suggested that the large differences between species required macro-mutations, a source of significant genetic changes (macro-evolution). According to Goldschmidt’s hypothesis, “one-step” macromutations in “controlling” genes might modify early development and cause profound and abrupt changes in the adult phenotypes of organisms that then acquire the potential to establish a new evolutionary lineage [41]. Obviously, the vast majority of macromutations could only be disastrous and evolution could proceed by the rare success of such hopeful monsters and not by the gradual accumulation of small changes within populations [42]. At the same time, C.H. Waddington (1905–1975) was trying to identify developmental mechanisms to produce such new species. To explain certain aspects of morphological evolution, he formulated the notion of transfer of competence, baptized the genetic assimilation, a process by which a phenotype originally induced in response to particular environmental conditions becomes encoded in the genome by natural selection or artificial selection [43]. Although controversial, the Waddington’s theory of genetic assimilation prefigured the notion of genome plasticity. In 1972, paleontologists N. Eldredge and S.J. Gould [44] provided a new perspective for the macroevolution model when they proposed the punctuated equilibrium theory as a complement to phyletic gradualism. This model proposed that biological evolution and the emergence of new species take place through abrupt and profound changes, occurring between long periods of stasis in which few variations occur in established species [45]. The punctuated changes might involve sporadic genetic mutations with large effects or chromosome rearrangements affecting gene expression. Punctuated equilibrium applies to sexually reproducing organisms and morphological evolutionary changes are regarded as largely correlated with speciation events [46]. The discovery of homeobox genes first in *Drosophila* and then in vertebrates has given rise to renewed interest in the concept of discontinuous evolution and macroevolution [47]. Phylogenic studies of the vertebrate *Hox* cluster and linked genes have suggested that the homeobox genes organization in malleable gene clusters occurred through processes of large-scale chromosomal rearrangements that reshape gene organization over evolutionary time [48, 49].

Many evolutionary events involve changes in the structure of chromosomes (fusion, translocation, inversion, insertion, ...) as evidenced by karyotype differences observed between mammalian species [50]. Heng [5] introduced the genome-centric concept in order to highlight the essential contribution of genome alterations (versus gene modifications) in the evolutionary adaptation and speciation. The genome-centric concept refers to the notion of macro-evolution proposed by Goldschmidt [42], and it allows to define tangible relationships between the biological self-organization and the natural selection [51]. One of the keys of the genome-centric evolutionary concept is the importance of the genome topology and karyotype, in connection with environment interactions, which can define an alternative conceptual framework, named the karyotype coding, for understanding genome beyond genes and fully appreciating evolutionary process [52, 53].

During the last years, experimental evidence has shown that genomic changes can confer a large adaptive value are not rare, and when competing with small-effect mutations, they tend to win [54, 55]. A growing number of studies has documented punctuated equilibrium and hopeful monsters in various species. For instance, Frazzetta [56] described a subfamily of monotypic snake, the *Bolyerinae*, distinct from all monotypic snakes in that they display a movable joint of the maxillary bone that constitutes a mechanical characteristic adaptive for feeding. Such a modification that arose suddenly, was in good agreement with the concept of the “Hopeful Monster”. Another example of discontinuous evolution concerns desert rodents. In 2 families of granivorous rodents (*Geomyidae* and *Heteromyidae*), Long [57] demonstrated that the formation of external check pouches conferring an adaptive benefit because they prevent salivary water loss to seeds, was not linked to a series of pre-adaptive possibilities but to a discontinuous transition. A striking example of such a short-term evolution was the fast adaptative changes of the lizard *Podarcis sicula* who, after 36 years of experimental introduction into a novel environment, displayed significant changes in morphology and performance (head morphology, bite strength) and a remarkable evolution of digestive tract structure [58]. In human, the neurobiological study of 2 subjects with six anatomically fully developed fingers on the two hands demonstrated the perfect cortical representation and control of supernumerary fingers and their augmented manipulation ability [59]. More anecdotal, the recent discovery of a python with a third functioning eye on its forehead could also be related to the macroevolution process (https://www.livescience.com/65382-three-eyed-snake.html). Similar ectopic induction of functional eyes has been genetically induced in scarab beetles [60]. The rapid emergence and integration of novel complex organs such as a eye or an extra finger provide remarkable examples of the ability of developmental systems to channel massive perturbations toward orderly and functional outcomes, highlighting the extraordinary plasticity of genome and the buffering capacity of developmental systems.

In 1999, Rieseberg et al. [61]introduced the notion of “transgressive segregations” for the generation and the rapid fixation of new genotypes in population. Based on genetic recombination in hybrids, this mechanism can rapidly produce novel phenotypes by recombining multiple loci simultaneously. If transgressive hybrids have higher fitness in environments, this increases the likelihood of divergence from the parental populations and of fixation in a population [62].

The phenomena of chromoanagenesis, by their sudden occurrence and the chaotic alterations of the genome that they generate, appear to be a mechanism consistent with these macroevolution concepts and the notion of punctuated change. Thus, genome chaos plays an essential role in the evolution of most cancers [63]. One-step catastrophic genomic events may generate multiple chromosomal rearrangements and significantly accelerate the tumor evolution [64–66]. In pancreatic cancer, the observation of a high prevalence of chromothripsis (> 65%) in tumors supports the punctuated equilibrium model as the principal process for tumorigenesis [67]. Among constitutional chromoanagenesis-related rearrangements, studies have evidenced that chromothripsis and chromoanasynthesis could generate massive but balanced genomic rearrangements, compatible with life and contributing or not to developmental diseases but also able to restore a normal function [68, 69]. To date, the existence of chaotic genomic alterations is not restricted to human but there are also documented in other mammalians [70], in plants [71, 72], in nematode *Caenorhabditis elegans* [73], in *Saccharomyces cerevisiae* [74] and plankton [75] emphasizing the notion that the cellular pathways responsible for generating such complex patterns of chromosomal rearrangements are highly conserved.

### Macro-evolutionary implications of chromoanagenesis

Undoubtedly, chromoanagenesis phenomena are among the most unexpected biological discoveries made in the last years. The investigation of this new class of genomic alterations has provided new insights on the mechanisms connecting their occurrence with cellular stress and genomic stability and integrity [76, 77]. A fundamental question is whether chromoanagenesis is a biological process that can promote the emergence of selective benefits in individuals that can be stably transmitted.

The hypothesis of such massive and abrupt changes doesn’t fundamentally discredit Darwinism and its general principle of microevolution. Indeed, macro-mutations could result from selection-driven responses to sudden environmental changes. They may serve as “key” adaption to shift its carrier toward a new mode of life. Sheldon [78] suggested that a punctuated equilibrium could prevail in unstable environments and gradualism in stable regimes. Theoretical models support the hypothesis that chromosomal rearrangements do play a key role in speciation in the face of gene flow. Chromosomal rearrangements could reduce gene flow through their suppressive effects on recombination rather than their effect on fitness [79, 80]. Chromoanagenesis-related rearrangements may promote rapid modifications in patterns of genes that are not related to recombination suppression, for instance by modifying gene position relative to replication origins, thus changing the mutational bias context. They also may lead to the generation of new gene linkage blocks or new chimeric genes as well as the disruption of the *cis-*regulatory machinery of gene expression [69]. The role of meiotic drive in the fixation of chromosomal rearrangements within the genome has also been demonstrated [81, 82].

The direct impact of chromosomal rearrangements on nuclear topology and gene expression is now studied directly through the analysis of regional genomic interactions by chromosome conformation capture techniques and the formation of topologically associating domains (TADs). These new approaches have made it possible to demonstrate in several pathologies how chromosomal rearrangements can disrupt TADS and affect the expression of the genes they contain by modifying or eliminating the interactions between promoters and enhancers [83–85]. An additional level of genomic regulation has been recently proposed with the description of a complex 3D network of well-delimited *cis* regulatory domains (CRDs) consistent with the chromatin organization into TADS, and in which 3D functional link and coordinated gene activity occurs along chromosomes [86].

The occurrence of genomic alterations and their impact are echoed in the notion of genome stability and the function of sexual reproduction as a filter to eliminate deleterious genomic changes, in maintaining the genome of a species [29, 52]. In this context, chromoanagenesis processes and the complex alterations which characterize them, could represent an important pathway for genome reorganization, and therefore for the emergence of new stable karyotypes, able to survive during evolution. Under high level of cellular stress, genome chaos and the subsequent occurrence of chromoanagenesis-mediated genome re-organization may constitute an effective survival strategy, by increasing karyotypic heterogeneity or/and creating new network in a given genome [53, 63]. The genome evolution process appears to be mainly based on the genome reshuffling rather than the accumulation of useful genes [52].

Accumulation of macromutations linked to chromoanagenesis events in gametogenesis or during early embryonic development could lead to profound differences among adults. Interestingly, de novo germline chromoanagenesis rearrangements predominantly occur on paternally derived chromosomes [7, 87]. Regardless of the beneficial or deleterious effect of complex chromosomal rearrangement, there is the question of its long-term fixation in a population. It is recognized that heterozygous carriers of chromosomal rearrangements can produce chromosomally unbalanced gametes in highly variable proportions, which can cause sterility and thus contribute to a form of reproductive isolation. As Gorelick and Heng [88] argued, sexual reproduction as a constraint on eukaryotic evolution, maintains ploidy and genome identity. However, over generations and on a large scale, this state of heterozygosity can turn into a state of homozygosity. Various models of chromosomal speciation refer to the existence of a gametic barrier resulting from the progressive fixation of one or more chromosomal rearrangements in a population [89, 90]. Such structural rearrangements that allow a genetic distinction between species are considered to be evolutionary rearrangements, and genome sequencing analyzes indicated that these evolutionary rearrangements were much more numerous than initially estimated [89]. For example, it was estimated in 1980 that the human and chimpanzee genomes differed only in 9 chromosomal inversions and one fusion [91]. In 2005, Newman et al. [92] identified 93 supplementary evolutionary rearrangements, ranging from 12 kb to 1 Mb. More than 245 large rearrangements including translocations, inversions, fusions, deletions, have been identified in the discriminative path between the mouse and humans [93], and numerous segmental duplications involved in human specific adaptive traits have been recently characterized by sequencing human and non-human primate genome [94]. Chromoanagenesis and genome instability may have given rise to rapid evolution in some mammalian species [95]. Crombach and Hogeweg [96] postulated that genome restructuring mediated by massive chromosomal rearrangements could be a beneficial operator for short-term adaptations to a new environment. They provide an efficient model that shows the evolution in gene ordering and clustering as a consequence of retrotransposon-mediated chromosome rearrangements. Transposable elements are recognized as significant contributors to chromosome evolution and speciation. Active and inactive transposable elements can serve as drivers in the formation of germline chromoanagenesis by compromising the genomic stability and facilitating chromatin conformation changes and DNA breaks [97]. They may promote adaptability of a population by generating changes in gene expression or promoting rapid chromosome restructuring [98, 99]. For instance, in the gibbon genome, the insertion of the retro-transposon LAVA in genes implicated in cell cycle progression and chromosome segregation appears to be at the origin of a high rate of chromothripsis-related rearrangements leading to the accelerated evolution of the gibbon karyotype and the emergence of different gibbon lineages, with highly rearranged chromosomes [70, 100]. Another example of speciation driven by massive chromosome rearrangements is the extensive chromosome reshuffling experienced by the marsupial family *Macropodidae*, with numerous interchromosomal rearrangements and diploid karyotype number ranging from 2n = 10 to 2n = 24 [101]. A great karyotypic variability is also observed in the *Arvicolinae* rodent family characterized by a high rate of complex intrachromosomal rearrangements and an important level of karyotypic evolution [102]. Finally, a parallel could be drawn between marker chromosomes generated by chromoanagenesis events and the supernumerary chromosomes, named B chromosomes, found in many eukaryotic karyotypes. It has been established that these supernumerary chromosomes represent multichromosomal mosaics arising in taxa that experienced rapid genome changes [103], like the domestic dog which has one of the most rearranged karyotypes in mammals [104, 105].

Finally, a fascinating emerging model, consistent with chromoanagenesis-related mechanisms, could be the punctuated occurrence of genomic rearrangements leading to the creation of new genes subsequently fixed by natural selection and contributing to diversity. The formation of functional and transmissible de novo genes from non-coding DNA has thus been described in various eukaryote lineages [106], clearly indicating the potential role of this phenomenon in adaptive evolution.

### Concluding perspective: towards a gradual acceptance of macroevolution

The identification of the catastrophic chromoanagenesis phenomena has modified our perception of the genesis and the aetiology of complex genomic rearrangements, but also on the extreme plasticity of genomes [107]. Integrity and stability of the genome are essential cellular objectives that chromoanasynthesis-related events can paradoxically contribute to maintain in case of cellular crisis, by the chaotic but sporadic reworkings of the genome that they can generate. As mechanism of rapid genome reorganization, chromoanagenesis plays an essential role in promoting macroevolution of genome and therefore might be regarded as a credible process for eukaryotic genome adaptation and speciation and the creation of new genetic networks during evolution.

With the development of new sequencing technologies, bioinformatics tools, genomic approaches and experimental evolutionary modelling, it becomes possible to explore genomes of various organisms, predict the evolution of reproducible patterns and to reconcile gradual and saltational evolutionary concepts [54, 108]. Even if gradual changes represent the common mode of evolution, punctuated and massive modifications have the potential to establish profound novelties sometimes facilitating adaptation. The impact of genome-level events emphasizes the need to establish new conceptual framework integrating the genome organization-based information [52, 53].

The integrative approach, called paleogenomics and combining cytogenetic maps, whole genome sequencing and genome studies, provides a new way to trace the evolutionary history of karyotypes, integrate the genome and genes relationships, and redefine the role of chromosome rearrangements in evolutionary processes [109].

The slow changes in the perception of the alternative phenomena of saltational evolution in the world of evolutionary biology show that the acceptance of the macroevolution remains a gradual process.

## Data Availability

The datasets used and analysed in this review are available from the corresponding author on reasonable request.

## Abbreviations

CCR: Complex chromosome rearrangement
DSB: Double-strand breaks
FoSTeS: Fork stalling and template switching
MMBIR: Microhomology-mediated break-induced replication
NHEJ: Non-homologous end-joining
TAD: Topologically associated domain
